# Training improved the note taking skill of nursing students in Aksum University; northern Ethiopia: a classroom-based action research

**DOI:** 10.1186/s13104-018-3614-0

**Published:** 2018-08-02

**Authors:** Awole Seid, Hafte Teklay

**Affiliations:** 1 Department of Nursing, Bahir Dar University, P.O. Box 79, Bahir Dar, Ethiopia; 2 Department of Biomedical Sciences, Aksum University, Aksum, Ethiopia

**Keywords:** Note taking, Motivation, Nursing, Students, Ethiopia

## Abstract

**Objective:**

Note taking is an effective strategy to improve students’ learning. It is considered that very few learners are fit enough for basic note taking skill. Thus, this study was aimed to assess note taking skill and motivation for learning of nursing students and to take action on the identified gaps.

**Results:**

The mean note taking skill score is 22.95 ± 4.766. The study demonstrates 9.1% of students had good note taking skill but 54.5 and 36.4% had moderate and poor note taking skills respectively. Regarding learning motivation, 13.6% had motivation and the rest 68.2 and 18.2% had moderate and poor motivation for learning to be a nurse respectively. On the items used to examine motivation, 54.1% of students were less motivated to ask questions in classroom though clarification is needed. Reasons for poor note taking showed 68.2 and 27.3% responded due to “most faculties are simply reading from the slides” and “students are confident that instructors will give slide copies later” respectively were the two main cited reasons respectively. Training nursing students about note taking techniques has made considerable impact on student’s learning behavior.

## Introduction

Note taking is advantageous to the students to understand lecture points and to improve recalling of information later. It increases class attention, active engagement in classes, clarification and paraphrasing of confusing points and their performance. Note taking is also useful as a reference material for a learner to review it when necessary [1].

It is considered that very few learners are fit enough for basic note taking skill. As taking notes is fast highlighting of information pointed out in lectures; it requires knowledge and skill to do so. Taking note involves condensed note of points, symbolization, relating to real life/environment and summarizing for easy memory of information [2].

Taking notes with students own words enable them to engage in all classroom activities. Various research studies on note taking produces evidences against all trends that lets students passive in classroom [3].

Research findings showed that there is strong and positive relationship between quality of note taking and academic performance. Not only in higher institution, note taking, can be successfully taught to children as young as elementary age. Moreover, many researchers assert that students can become more effective note takers by receiving systematic note taking training by experts, and by practicing and comparing their own notes with peers’ [4].

The poor quality of student notes may reflect not only a lack of skills necessary to take accurate and complete notes but also the complexity of the task. Note taking involves listening to new and often unfamiliar information, transcribing that information quickly enough to keep pace with the lecture, and deciding how to organize the material to reflect the relationships stated by the speaker [5]. Several studies indicate that students have difficulty organizing lecture material and identifying main points. Furthermore, students say they experience the most difficulty with lecturers that speak too quickly or inaudibly, fail to present a clear outline of the beginning of the lecture, or fail to signal important information [6].

Instructors who thought nursing students in Aksum University complained about a gap on note taking during lecture. As the same time, students often failed the exam if the exam questions are extracted out of the handout/reading material given but discussed in the classroom. In addition, students look passive in class even if frequent questions are forwarded. One of our research team member explained students were following him like “film actor” while lecturing. He means they did not take notes at all.

This is very challenging on the students’ academic success that in turn would negatively affect student’s competency of learning. Thus, this action research is conducted aiming at improving the note taking skill of nursing students and contribute in enhancing students academic performance. One of the means of achieving this is through action research defined as a systematic research process that can be articulated by the researcher, involving data collection and analysis as well as reflection and discussion with co-researchers or others for the purpose of making change in situation overtime. Even though the early nursing theorist Betty Newman advocated early for use of action research in nursing due to the direct connection to practice, limited to use is evident in nursing education [7].

## Main text

### Methods and materials

The study was conducted at third year nursing department students in Aksum University on March, 2016. The tool used to assess note taking skill and motivation is adopted from university of Houston counseling service (webmaster@uhcl.edu). The questionnaire has two parts. Note taking and motivation. Each part has 8 questions with a maximum score of 32. A self-administered anonymous questionnaire was prepared and distributed for each student. Data is analyzed using SPSS version 20. The result is described using texts, tables and graphs as necessary. The association between variables was measured using correlation (r) as it is appropriate for two continuous exposure and outcome variables. Moderate correlation is present if r > 0.5 and statistically significant if the p value is < 0.05.

#### Operational definition

- Good note taking skill: students score 29–32 points.
- Moderate note taking skill: students score 21–28 points.
- Poor note taking skill: students score below 20.
- Good motivation: students score 29–32.
- Moderate motivation: students score 21–28.
- Poor motivation: students score below 20.

### Result

From a total 30 students 22 are participated in the study. The response rate is 100%. The major findings of the study are presented as follows.

From the study participants only one student is female and the rest were males. The level of perceived academic performance shows 16 (72.7%) perceive as medium level and 6 (27.3%) as high performance but none of them reported as poor achievers.

#### Note taking skill results

The mean note taking skill score is 22.95 ± 4.766. The minimum and maximum scores students get in note taking skill is 13 and 31 respectively.

**Fig. 1:**
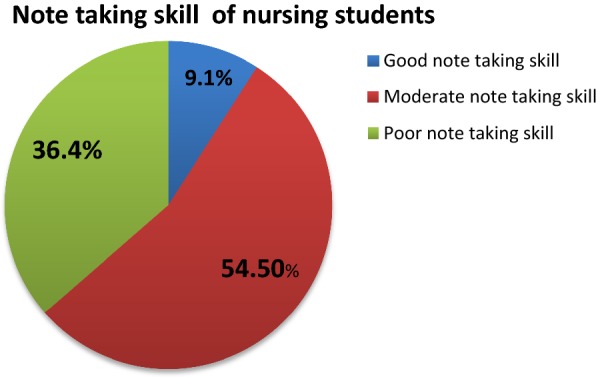
Distribution of note taking skill of 3rd year nursing students, Aksum University, Northern Ethiopia, 2016

As shown above, from the total study participants 36.4% have poor note taking skills and only 9.1% have good note taking skills (Fig. 1).

Among the sub questions presented to assess note taking skills of students, students have relatively good behavior with regard to taking notes using outline format except for mind maps (45.5%) and a good culture of taking notes while they read supplementary readings (40.9%). On the other hand, students have poor behavior with regard to “understanding lecture while taking notes during classroom time” and “reviewing notes after class or during the night time”.

Finding on reason for poor note taking skill showed, 68.2 and 27.3% responded due to “most faculties are simply reading from the slides” and due to “students are confident that instructors will give them hard copy slide prints later” are the two main cited reasons respectively.

#### Motivation level results

In this regard, the mean motivation score among the study participants are 24 ± 4.071 and the minimum and maximum motivation for learning score is 13 and 16 respectively. On the items used to examine motivation, 54.1% of students were less motivated to ask questions in classroom though clarification is needed.

**Fig. 2:**
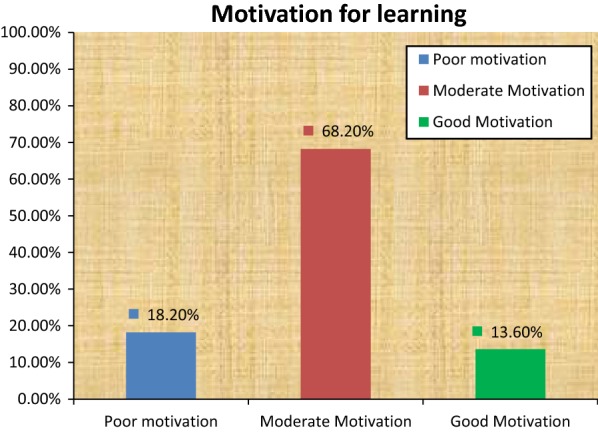
Distribution of Motivation status of 3rd year nursing students, Aksum University, Northern Ethiopia, 2016

The finding on motivation reveals 18.2% have poor motivation and 13.6% have good motivation for learning in the classroom (Fig. 2).

Correlation finding showed there is statistically significant correlation between motivation and note taking skill of students (r = 0.663, p = 0.001). This also implies that 43.95% of the variation in score of note taking skill is explained by the difference in motivation status of students (r^2^ = 0.439) (Table 1).

**Table 1:** Action plan to improve note taking skill of nursing students, Aksum University, northern Ethiopia, 2016

Problem	Activity	Responsible body	Time frame	Resources	Monitoring and evaluation strategy
Poor note taking skill	Training on note taking skills Discontinuing giving handouts for courses with adequate references Advising instructors to minimize slides and prepare in advance before class	Research team members Instructors Nursing department	18/4/2016, 2.00 p.m. Starting from next year (2017)	LCD projector, tea break, flip chart, marker Books Modules	Observing change of behavior in consecutive classes after training Including questions that are not given by handout but discussed in classroom

#### Implementation

On 18/4/2016 at 2.00 p.m. the research team members gave training for 3rd year nursing students about note taking skills. After tutors’ give an essay like materials, students were grouped and enabled to do exercises on note taking skill using different techniques. One group presented notes using outline format, another using table, and the remaining using a mind map. Comments were given from peers and HDP candidates as well.

The study finding is presented to the department staffs and reached consensus on discontinuing giving handouts for courses with adequate references and to minimize the number of slides. Additionally, instructors should prepare in advance and promote students engagement in class by transferring the responsibility for learning to them.

#### Evaluation

As we gather from the instructor more students are taking notes in classroom. The students need continuous monitoring for the coming year too and hoping that discontinuing handouts will provoke them to take notes in classroom and while reading books.

The intervention promotes students for independent learning (self-directed learning) through engagement in classroom and the students highlight main points in class and further expand their knowledge through reading different references. The intervention aids student to exercise time saving technique of note taking during lecture time and reading texts. After continuous evaluation if the problem is recurring the study may be repeated and other ways interventions will be designed.

### Discussion

This study provides a glance view of the note taking skills status of nursing students with small sample size. The implications of this research finding on practice suggest there will be potential impact on quality of health care at the end unless promptly intervened.

The finding on note taking skill also suggests the need to adopt experiences from local and other international perspectives. As most students face difficulty of inquiring at classroom the university should cultivate the value of freedom of speech in schools. Modification of interventions may required if the study produces similar result at college level like adopting as a policy to train students about note taking and other necessary skills may be necessary. Furthermore, conducting classroom based action research should be one of educational responsibility of faculties.

### Conclusion

Note taking facilitates both recall of factual material and the synthesis and application of new knowledge, particularly when notes are reviewed prior to exams. It enables students to actively engage in learning process. Creating awareness about the benefit of note taking and training students about different techniques of note taking has made considerable impact on student’s learning behavior.

## Limitations

The limitation of this study is small sample size and the impact on training wasn’t assessed continuously. Therefore, studying with large sample size on longitudinal method is recommended.

## Abbreviations

HDP: Higher Diploma Program
SPSS: Software Program for Social Sciences
